# Binaural Beats through the Auditory Pathway: From Brainstem to Connectivity Patterns

**DOI:** 10.1523/ENEURO.0232-19.2020

**Published:** 2020-03-11

**Authors:** Hector D. Orozco Perez, Guillaume Dumas, Alexandre Lehmann

**Affiliations:** 1 Laboratory for Brain, Music and Sound Research (BRAMS), Montreal H2V 2S9, Canada; 2 Department of Psychology, Neuroscience and Behavior, McMaster University, Hamilton L8S 4L8, Canada; 3 Human Genetics and Cognitive Functions, Institut Pasteur, Unité Mixte de Recherche 3571 Centre National de la Recherche Scientifique, Université de Paris, Paris 75015, France; 4 Center for Complex Systems and Brain Sciences, Florida Atlantic University, Boca Raton, FL 33431; 5 Department of Otolaryngology Head and Neck Surgery, McGill University, Montreal H3A 0G4, Canada; 6 Centre for Research on Brain, Language and Music (CRBLM), Montreal H3G 2A8, Canada

**Keywords:** binaural beats, brain connectivity, brain entrainment, EEG

## Abstract

Binaural beating is a perceptual auditory illusion occurring when presenting two neighboring frequencies to each ear separately. Several controversial claims have been attributed to binaural beats regarding their ability to entrain human brain activity and mood, in both the scientific literature and the marketing realm. Here, we sought to address those questions in a robust fashion using a single-blind, active-controlled protocol. To do so, we compared the effects of binaural beats with a control beat stimulation (monaural beats, known to entrain brain activity but not mood) across four distinct levels in the human auditory pathway: subcortical and cortical entrainment, scalp-level functional connectivity and self-reports. Both stimuli elicited standard subcortical responses at the pure tone frequencies of the stimulus [i.e., frequency following response (FFR)], and entrained the cortex at the beat frequency [i.e., auditory steady state response (ASSR)]. Furthermore, functional connectivity patterns were modulated differentially by both kinds of stimuli, with binaural beats being the only one eliciting cross-frequency activity. Despite this, we did not find any mood modulation related to our experimental manipulation. Our results provide evidence that binaural beats elicit cross frequency connectivity patterns, but weakly entrain the cortex when compared with monaural beat stimuli. Whether binaural beats have an impact on cognitive performance or other mood measurements remains to be seen and can be further investigated within the proposed methodological framework.

## Visual Abstract

**Figure:**
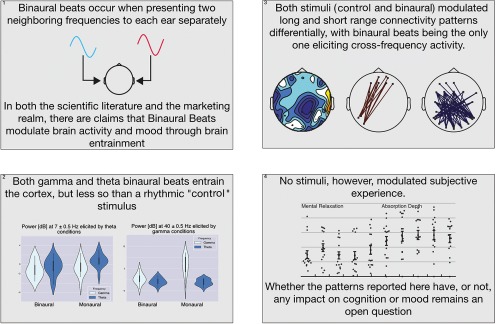

## Significance Statement

Binaural beats have been a source of speculation and debate in the scientific community. Our study addresses controversial claims and approaches them using proper experimental control and modern signal processing techniques. Here, we show that binaural beats can both entrain the cortex and elicit specific connectivity patterns. Regardless of this, our monaural control condition was able to entrain the cortex more strongly, and both binaural beats and the control condition failed to regulate mood. All in all, though binaural beats entrain cortical activity and elicit complex patterns of connectivity, the functional significance (if any) of binaural beats, and whether they are more “special” than monaural beats remain open questions.

## Introduction

Humans use music and rhythm as mood enhancers. Be it in social gatherings or late study nights, we use audio stimuli to set the “right mood” and improve our cognitive performance (Mammarella et al., 2007; Schellenberg et al., 2007; Tarr et al., 2014). Binaural beats, an auditory illusion that occurs when presenting two similar pure tones to each ear separately, have been purported to induce mood alterations, contingent on the beat frequency. Claims range from entraining the whole brain (Atwater, 2004; Rhodes, 1993), to altering states of consciousness (I-Doser, accessed May 2018; Atwater, 1997). The possibility of binaural beats modulating cognitive states without prior training makes them an interesting candidate for cost-effective applications in both healthy and impaired populations.

Presenting two tones with a slight frequency mismatch to each ear separately creates a perception of a third tone, a binaural beat, that oscillates at the absolute difference between the tones (Oster, 1973; Moore, 2012). These beats are thought to originate subcortically in the medial nucleus of the superior olivary complex, the first nucleus in the auditory pathway to receive bilateral input (Wernick and Starr, 1968; Kuwada et al., 1979). This “illusory” third tone is lateralized between the left and right ear of the listener, making binaural beats useful for spatial sound research (Ross et al., 2014). Binaural beats can entrain cortical activity at both the specific frequency of the beat (Pratt et al., 2010) and cross-frequency modulations, such as θ beats driving interhemispheric α synchronization (Solcà et al., 2016). They also seem to modulate mood (Wahbeh et al., 2007), pain perception (Zampi, 2015), and cognitive performance in memory tasks (Kennerly, 1994). The cognitive effects of binaural beats are attributed to their capacity to drive neural oscillations at the beat frequency through differential hemispheric synchronization frequencies. The reported cognitive modulations, however, appear inconsistent and seem to depend on several mediating factors, such as frequency of stimulation, differing exposure time and stimuli masking (Garcia-Argibay et al., 2019a). Furthermore, no study to date has fully characterized binaural beats throughout the auditory pathway (from subcortical responses to functional connectivity) and compared their effect to that of a non-binaural rhythmic control (i.e., monaural beats, created by digitally summing each tone before presentation). Indeed, monaural beats readily entrain the cortex to specific frequencies (Nozaradan et al., 2016), and repetitive and rhythmic stimuli (such as mantras or tones) are widely used in contemplative and religious practices with positive physiological impact (Bernardi et al., 2001, 2017). It remains open questions whether the reported effects of binaural beats are due to: (1) their asymmetrically driven rhythmicity (the binaural aspect is essential to their effectiveness), (2) their capability of entraining brain oscillations (as would also be the case with monaural beats), or (3) a placebo effect. To address these, we recorded electroencephalography (EEG) during a single-blind, active-controlled task in which participants listened to both binaural and monaural beats.

Our main objective was to characterize brain responses and cognitive alterations induced by binaural beats, compared with a monaural beat control condition. Our secondary objective was to compare the neural and subjective effects elicited by two different beat frequencies. We used θ (7 Hz) beats because they have been associated with reduced anxiety levels (Isik et al., 2017), and γ beats (40 Hz) because they have been associated with attention modulation (Colzato et al., 2017). Furthermore, these frequencies have been associated with large-scale integration models of the brain (Varela et al., 2001; Canolty and Knight, 2010). We compared responses between binaural and monaural beats at four levels: subcortical entrainment to the carrier tones in the form of a frequency following response (FFR; Skoe and Kraus, 2010), cortical entrainment to the beat in the form of an auditory steady state response (ASSR; Picton et al., 2003), changes in functional connectivity using phase-based statistics (Nolte et al., 2004; Lachaux et al., 2000) and self-reported mood changes using analogue scales (Rainville et al., 2002). We hypothesized both beats would elicit cortical and subcortical responses to the beat (ASSR) and pure tone frequencies (FFR), respectively. However, we expected binaural beats to elicit functional connectivity changes and modulate mood, with no such changes during the control condition. We hypothesized that θ beats would facilitate a relaxed state, while γ beats would elicit a more alert state. By presenting converging evidence from different approaches (self-reports, EEG), we aimed to elucidate whether binaural beats are a special kind of stimulus or reported effects could be achieved with non-binaural rhythmic stimuli.

## Materials and Methods

To understand the functional meaning of the entrainment and connectivity patterns associated with binaural beats, we investigated the differences between monaural and binaural beats by comparing subcortical, cortical and subjective responses elicited through a single-blind, passive listening task with a 2 × 2 factorial design (two within factors: beat type and beat frequency).

### Participants

Sixteen participants (nine female, seven male; mean age 27.4 ± 5.5) volunteered for the experiment and provided written informed consent. Exclusion criteria included neurologic damage or abnormalities (e.g., demyelination), and major hearing loss (0–20 HL dB) as self-reported by the participants. The experimental procedures conformed to the World Medical Association’s Declaration of Helsinki and were approved by the Research Ethics Committee of the Faculty for Arts and Sciences of the University of Montreal.

### Stimuli

**Figure 1.**
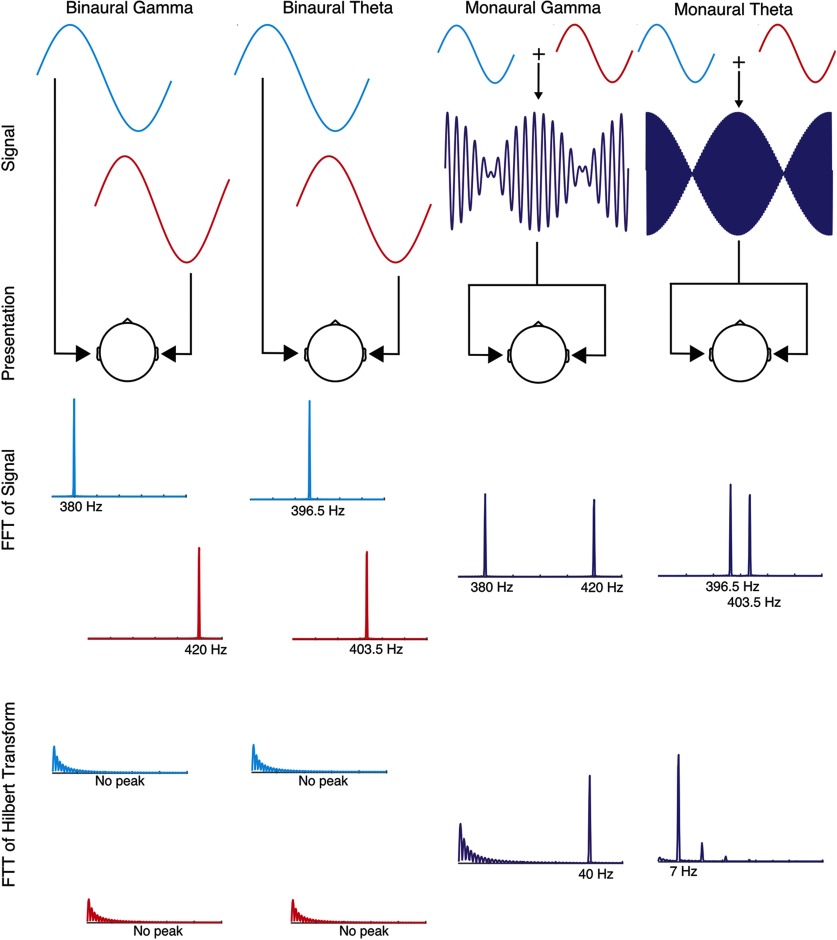
Beats: Signal, Presentation Method, Fast Fourier Transform, FFT of Hilbert Transform. Each column represents one experimental condition. Signal and presentation rows, Binaural beats are created by dichotically presenting two pure tones with a slight frequency mismatch (red color = right ear). Monaural beats are created by digitally summing these tones and presenting the resulting signal diotically. FFT of signal, Stimuli were analyzed using a Fourier transform to obtain their frequency composition. FFT of Hilbert transform, The FFT of the Hilbert transform (i.e., the analytic signal) was computed to tap into the spectral information of the envelope of the signal (the beat frequency). The frequency of the envelope of the summed tones encodes beat frequency (e.g., 403.5–396.5 = 7 Hz for θ). This information, however, is only encoded in monaural beats because they are digitally summed.

Binaural beats entrain cortical activity at the specific frequency of the beat percept (Schwarz and Taylor, 2005; Draganova et al., 2008; Pratt et al., 2010). We refer to beat frequency as the frequency of this percept, which is the difference between the pure tones; either 40 Hz for γ conditions (380 and 420 Hz) or 7 Hz for θ conditions (396.5 and 403.5 Hz). This rhythmic percept is a key piece of the purported subjective effects of binaural beats in the scientific and pseudoscientific literature. They claim entrainment to it regulates mood and cognition. To elucidate whether and how binaural beats regulate mood, we chose monaural beats as a control that would rule out rhythmicity as an influencing factor. Binaural beats do not contain the beat frequency in neither their spectrum nor their envelope, but this percept is presumably created in the medial nucleus of the superior olivary complex (Wernick and Starr, 1968; Kuwada et al., 1979). On the other hand, monaural beats do contain it in their envelope (Fig. 1).

Binaural beat stimuli consisted of two pure sine tones with equal starting phase and a slight frequency mismatch presented separately to each ear (Fig. 1, columns 1 and 2). These two pure tones were superimposed digitally (added together and divided by two to control for loudness) to create the monaural control condition, which was presented monaurally to both ears, each ear was presented with the same stimuli (for other examples where monaural beats were used as a control condition, see Schwarz and Taylor, 2005; Draganova et al., 2008; Becher et al., 2015). By summing both pure tones, we essentially created amplitude-modulated stimuli, which excel at entraining the brain (Ross et al., 2000).

We chose carrier frequencies around 400 Hz for two reasons: best perception of binaural beats occurs at carrier tones between 400 and 500 Hz (Licklider et al., 1950; Perrott and Nelson, 1969), and this frequency range minimizes cortical contributions to the brainstem responses (Coffey et al., 2016). Both kinds of stimuli (binaural and monaural control) were root mean squared (rms) normalized. The lower of both frequencies (380 and 396.5 Hz) was always presented to the left ear (i.e., pure tone presentation was not altered between left and right ears).

### Frequency choice: θ

Auditory stimulation at the θ frequency band (4–7 Hz) has been associated with positive emotional experiences and introspection (Aftanas and Golocheikine, 2001), reduced perceived pain in patients with chronic pain (Zampi, 2015), states of meditation and decreased alertness (Jirakittayakorn and Wongsawat, 2017), and enhancement of immediate verbal memory (Ortiz et al., 2008). Furthermore, θ cortical activity is related to concentration, focused attention and a general meditative state (Takahashi et al., 2005; Lagopoulos et al., 2009). We chose θ beat frequency to explore the possibility of eliciting a mindful and relaxed state in the participants.

### Frequency choice: γ

Auditory γ stimulation (32–48 Hz) has been associated with binaural sound integration (Ross et al., 2014), divergent thinking (Reedijk et al., 2013), and attention control (Reedijk et al., 2015). Furthermore, auditory cortices readily entrain to it (Schwarz and Taylor, 2005; Ross et al., 2014), and it seems to be a “natural frequency” of these areas, even during resting state (Hillebrand et al., 2012). We chose γ beat frequency to explore the possibility of eliciting a heightened attention cognitive state in the participants.

### Procedure

Participants started by filling out a general information and music abilities questionnaire. We then fitted a headcap on participants’ heads and placed EEG electrodes in it using a conductive gel. The experiment took place in a sound-attenuated, electromagnetically shielded room. Participants were asked to relax their upper body, close their eyes, avoid body movements and to pay attention to the beat throughout the experiment (Schwarz and Taylor, 2005). We recorded data from five experimental blocks: an 8 min baseline (no stimulus presentation; eyes-closed) followed by the four pseudorandomized experimental conditions (binaural γ, monaural γ, binaural θ, monaural θ), each lasting for 8 min. After each recording block, participants were asked to rate their experience using two visual analogue scales. They were also given the opportunity to take a break in the middle of the experiment. Auditory stimuli (both binaural and monaural beats) were generated live (i.e., during the recording block) to ensure submillisecond phase accuracy using a signal processing system (RX6, Tucker-Davis Technologies) controlled with MATLAB software (The MathWorks) and delivered via insert earphones (ER3, Etymotic Research). Auditory stimuli were processed at 48 kHz and were each presented continuously for 8 min at 70-dB SPL. For the purpose of further analysis and the epoching of continuous data, triggers were sent every 8 s via parallel ports using the signal processing system (RX6, Tucker-Davis Technologies) and recorded along with the EEG data.

### Sound calibration

Output sound from the signal processing system was calibrated to be presented at 70-dB SPL at the level of each ear, using a Sound-Pro sound level meter (model DL 1/3 Octave Datalogging RTA) and a 2-CC ear coupler for insert earphones calibration. Calibration measurements were done using a slow rate mode with an A-weighting frequency filter.

### EEG data acquisition

EEG was recorded using 64 active sintered Ag-AgCl electrodes placed on the scalp according to the International 10/10 system (ActiveTwo, BioSemi). The active electrodes contain the first amplifier stage within the electrode cover and provide impedance transformation on the electrode to prevent interference currents from generating significant impedance-dependent nuisance voltages. We, therefore, did not control electrode impedances but rather kept direct-current offset close to zero during electrode placement. Vertical and horizontal eye movements were monitored using three additional electrodes placed on the outer canthus of each eye and on the inferior area of the left orbit. Reference-free electrode signals were amplified, sampled at 2048 Hz (ActiveTwo amplifier, BioSemi), and stored using BioSemi ActiView Software for offline analysis. Given that auditory stimuli were created online during the experiment, they were recorded using BioSemi’s. Analog Input Box (BioSemi), which was daisy chained by optical fibers to the EEG Analog-to-Digital Converter box and stored alongside the EEG data for future analysis.

### Visual analogue scales

**Figure 2.**
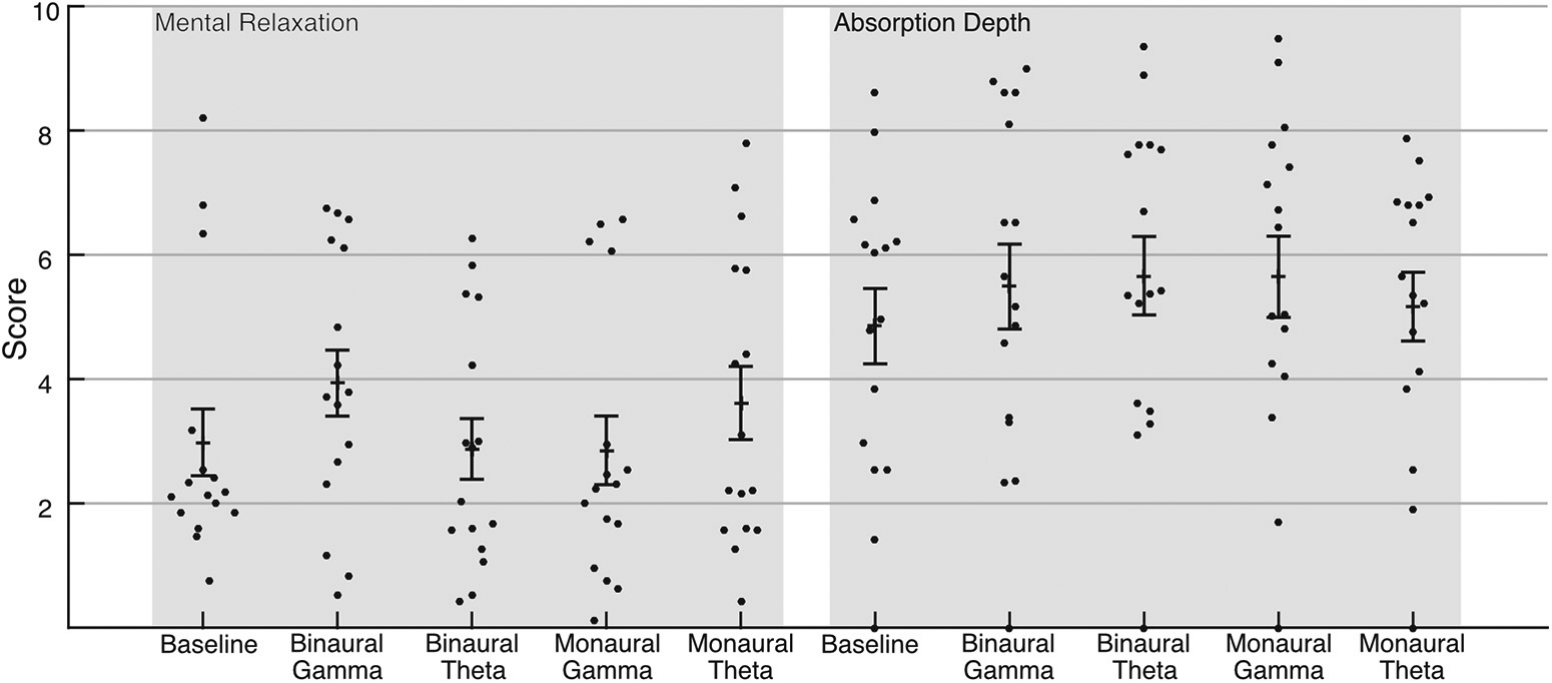
Visual analogue scales. Mental relaxation and absorption depth. Each data point represents one participants’ self reported score. Mean is plotted as a black horizontal line ± standard error of the mean (SEM).

Participants were given pen and paper analogue scales after each recording block so they could rate their experience after the passive listening task. Two analogue scales were used to determine variations in subjective experience (Rainville et al., 2002). The scales used were the following. (1) Mental relaxation, corresponding to the activity or calmness of the subject’s mind. This dimension spans from a state where the mind is calm, peaceful and in perfect relaxation to a state where the mind is extremely agitated or active. (2) Absorption depth corresponds to how the subject feels and how absorbed they felt during the experiment (Fig. 2). The scale runs from nonexistent depth to a profound, intense and complete experience.

### Data analysis and signal processing

#### Software accessibility

All the code used for this project (digital signal processing, data wrangling, and statistics) can be found here: www.github.com/neurohazardous/binauralBeats.

#### Visual analogue scales

Data from pen and paper scales was measured manually and stored digitally in CSV files for further statistical analysis using R (v3.6.1, R Development Core team, 2008), setting the significance level at 0.05. We first determined the data distribution using a Shapiro–Wilk test. Wherever data were not normal, the specified statistic was compared against a distribution created by permuting the data 1000 times (i.e., scrambling the label of the data), as opposed to comparing the statistic against a parametric distribution (Ernst, 2004). We also report confidence intervals obtained from the null distribution (obtained by permuting the data). Given the within-nature of our study, we only performed permutations within-subjects (e.g., if data were arranged in a matrix were each row is one subject and each column is a measurement, we only permuted the labels of the values within each row). The reported *p* value was obtained as the number of permuted statistics that were larger than the specified statistic, divided by the total number of permutations. We analyzed the data using a one-way repeated measures ANOVA for each scale (mental relaxation and absorption depth) with “condition” as a five-level factor (baseline, monaural γ, binaural θ, binaural γ, monaural θ). We used *post hoc* paired *t* tests to further disentangle patterns in the data only when the F statistic reached significance. We kept the Family-wise error rate (FWER) at *p* = 0.05 by using Holm’s sequential Bonferroni procedure (Table 1).

**Table 1.** Statistical table

	Data structure	Statistical test	C.I.
a	Not normal (*W* = 0.9, *p* = 0.0001)	Permutation one-way repeated measures ANOVA	0.13, 3.04
b	Normal (*W* = 0.96, *p* = 0.013)	Permutation one-way repeated measures ANOVA	0.09, 3.08
c	Normal (*W* = 0.99, *p* = 0.86)	Permutation factorial (2 × 2) repeated measures ANOVA Frequency Beat type Interaction	0.001, 5.6 0.002, 5.99 0.001, 5.67
d	Not normal (*W* = 0.96, *p* = 0.05)	Permutation factorial (2 × 2) repeated measures ANOVA Frequency Beat type Interaction	0.001, 5.46 0.001, 6.1 0.0003, 5.45
e	Not normal (*W* = 0.492, *p* = 1.549e-13)	Permutation factorial (2 × 2) repeated measures ANOVA Frequency Beat type Interaction	0.001, 6.31 0.001, 6.66 0.001, 6.43
f	Not normal (*W* = 0.625, *p* = 1.631e-11)	Permutation factorial (2 × 2) repeated measures ANOVA Frequency Beat type Interaction *Post hoc* permutation paired *t* test (monaural γ vs monaural θ) *Post hoc* permutation paired *t* test (binaural γ vs monaural γ) *Post hoc* permutation paired *t* test (binaural γ vs binaural)	0.001, 7.1 0.001, 7.68 0.001, 6.26 –2.02, 2.39 –2.4, 2.23 –2.1, 2
g	Spatial-spectral-temporal (Hilbert Transform)	Non-parametric, cluster-based permutation tests Monaural γ vs baseline at 40 Hz	0.57, 4.07
h	Spatial-spectral-temporal (PLV)	Non-parametric, cluster-based permutation tests Binaural θ vs monaural θ at 40 Hz Binaural γ vs monaural γ at α Binaural γ vs monaural γ at γ Binaural γ vs monaural γ at γ Binaural γ vs monaural γ at 40 Hz Monaural γ vs baseline at 40 Hz	2.55, 5.99 2.02, 6.04 2.5, 6.11 2.5, 6.11 2.69, 6.62 2.79, 6.99
i	Spatial-spectral-temporal (Fourier transform)	Non-parametric, cluster-based permutation tests Binaural θ vs baseline at θ Binaural θ vs baseline at 40 Hz	0.85, 4.89 –1.39, 4.06
j	Spatial-spectral-temporal (neurophenomenological)	Non-parametric, cluster-based permutation tests MR, Negative frontal cluster at θ MR, negative frontocentral to occipital at θ MR, Negative frontal to right temporal occipital at 40 Hz AD, Negative right temporal at 40 Hz	0.93, 3.25 3.2, 6.38 2.54, 5.86 –1.45, 4.03

Description of statistical tests and confidence intervals (C.I.) for each of the results reported on the main text.

#### EEG

The data were processed using the EEGLAB toolbox (Delorme and Makeig, 2004) and in-house developed scripts in MATLAB. Two different analyses were conducted on the raw EEG data: subcortical (FFR) and cortical (ASSR and functional connectivity). The pre-processing procedures for either subcortical or cortical analysis differed in filtering process, ICA decomposition and re-referencing. For subcortical analysis, data were high-pass filtered at 100 Hz and re-referenced to linked mastoids. Data used for cortical analysis was bandpass filtered between 1–100 Hz, decomposed using ICA for artifact correction purposes (Jung et al., 1998) and re-referenced to linked mastoids as a first step and then to common average reference as a final step.

#### FFR

Data were re-referenced to linked mastoids and high-pass filtered at 100 Hz using a zero-phase Butterworth filter Order 4. Data were visually inspected for noisy electrodes, which were then removed and interpolated using spherical interpolation. Finally, data were epoched into 60 events (from −1 to 7 s with respect to trigger onset) and exported for further analysis. Epochs from each participant were averaged and transformed into the frequency domain using an fast Fourier transform (FFT). From these, power was calculated as the square of the magnitude normalized using a factor of *2/N*, *N* being the length of the epoch. Frequencies of interest were extracted as the mean of 1-Hz bins around the carrier frequencies (pure tones: 380, 397.5, 403.5, and 420 Hz) for the baseline and each experimental condition. A baseline normalization (decibel change from baseline) was performed to disentangle background dynamics from actual stimulation-related oscillations (Cohen, 2014). The equation used was as follows:

(1) $$dB_{f}=10log_{10}\left(\frac{activity_{f}}{\bar{baseline_{f}}}\right),$$

where $activity_{f}$ is a specific frequency power in a given experimental condition and $\bar{baseline_{f}}$ is the average activity across the whole baseline at a given frequency (Cohen, 2014). The unit of these data is decibel change from baseline.

After baseline normalization, all the scalp channels were averaged together to output one normalized power score per experimental condition per participant. Frequency relevant scores were averaged together in each experimental condition. For example, power scores for 396.5 and 403.5 Hz were averaged together for θ frequency relevant scores. This was done in order to keep the hypothesis testing at a minimum and avoiding inflating the FWER. These averaged scores were then exported to R (v3.6.1, R Development Core team, 2008) for hypothesis testing.

We first determined the data distribution using a Shapiro–Wilk test setting the significance level at 0.01. Wherever data were not normal, the specified statistic was compared against a distribution created by permuting the data within each participant for 1000 times (Ernst, 2004) and comparing the specified statistic against this distribution. As with the VAS, we report two-tailed confidence intervals (0.95%) obtained from the null distribution. Two sets of data were analyzed (power at relevant γ and relevant θ frequencies) with 64 scores each (four conditions × 16 participants) for statistical significance. A factorial (2 × 2) repeated measures ANOVA was computed per relevant pure tone data set (θ and γ) using beat type (binaural, monaural) and frequency (γ, θ) as within factors. When the interaction between the factors was significant, we calculated *post hoc* paired *t* tests to further disentangle patterns in the data (i.e., identify which experimental condition elicited the highest response). We used Holm’s sequential Bonferroni correction to keep the FWER at 0.05 (Table 1).

### ASSRs

Data were imported and re-referenced to linked mastoids. Data were then resampled at 512 Hz, trimmed around the time-window of interest (8 m ± 3 s) and filtered twice: using a second order Butterworth bandpass filter (zero-phase) between 1 and 100 Hz, and an FIR notch filter at 60 Hz (minimizing line noise). Data were visually inspected for noisy electrodes, which were then removed.

For each participant, ICA decomposition was applied to the full recording of all conditions. Prior to this, these aggregated files were first filtered between 1 and 80 Hz and decimated to 256 Hz. Data were then decomposed using the *runica()* function from EEGlab, which uses the Bell and Sejnowski (1995)’s ICA algorithm and Lee, Girolami, and Sejnowski’s extended-ICA algorithm (Lee et al., 2000). After visual inspection of individual components, weight matrices were obtained from this decomposition and applied to the original five files for artifact correction purposes (remove components deemed as non-cortical activity; Jung et al., 1998). Missing electrodes were interpolated after ICA artifact correction. EEG was re-referenced to common average (CAR) and epoched from –1 to 8 s relative to trigger onset. Finally, data were baseline corrected (using whole epoch as the baseline) and stored for further analysis.

Each participant’s epochs were averaged and transformed into the frequency domain using an FFT. From these, power was calculated as the square of the magnitude normalized using a factor equal to 2/*N*, where *N* is the number of samples in each sequence. Frequencies of interest were extracted as the mean of 1-Hz bins around beat frequencies (7 and 40 Hz) for both the baseline and each experimental condition. As with the FFR preprocessing procedure, a baseline normalization (decibel change from baseline) was done using Equation 1.

After baseline normalization, all channels were averaged together to output one normalized power score per experimental condition per participant. These scores were then exported to R (v3.6.1, R Development Core team, 2008) for hypothesis testing.

Statistical analyses were very similar to those done for the FFR analysis. We first determined the data distribution using a Shapiro–Wilk test. Wherever data were not normal, the specified statistic was compared against a distribution created by permuting the data within each participant for 1000 times (Ernst, 2004) and comparing the specified statistic against this distribution. We report two-tailed confidence intervals (0.95%) obtained from this null distribution. Two statistical analysis were performed: one on normalized power scores at γ beat frequency and one on normalized power scores at θ beat frequency (each had 64 scores total, four conditions × 16 participants). Hypothesis testing was performed using a factorial (2 × 2) repeated measures ANOVA with beat type (binaural, monaural) and frequency (γ, θ) as within factors. Finally, *post hoc* paired *t* tests were calculated wherever there was a significant interaction between factors to identify which experimental conditions elicited the highest entrainment. We used Holm’s sequential Bonferroni procedure to keep the FWER at 0.05 (Table 1).

### Functional connectivity

Two complementary measurements of functional connectivity were used as indices of long-range synchronization: phase-locking value (PLV; Lachaux et al., 2000) and imaginary coherence (iCOH; Nolte et al., 2004). On top of that, per electrode, the amplitude of the Hilbert Transform and the Power of the Fourier transform were computed as local indices of synchronization. Analyses were done over all traditional frequency bands (δ: 1–4 Hz; θ: 5–8 Hz, α: 9–12 Hz, β: 13–30 Hz, γ: 32–48 Hz) and the specific beat frequencies (1-Hz bins around 7 and 40 Hz). ICA corrected data (i.e., the same files used for ASSR) was imported to MATLAB to compute these metrics.

### Hilbert transform and PLV

The PLV looks at how stable phase differences are between signals (in this case, electrodes). In this particular implementation, it determines, on average, how stable phase differences between electrodes are within trials (i.e., over time). PLV is only sensitive to phase differences between signals (not their amplitude) at the cost of not being able to distinguish spurious correlation due to volume conduction at the scalp level from actual connectivity between two cortical regions (Lachaux et al., 2000).

To calculate it, the signal of interest was extracted by band-passing the ICA corrected data using a finite impulse response filter (FIR) around both the traditional frequency bands of interest and the specific beat frequencies: δ (1–4 Hz), θ (5–8 Hz), α (9–12 Hz), β (13–30 Hz), γ (32–48 Hz), θ Beat (6–8 Hz) and γ Beat (39–41 Hz). Phase and amplitude of the analytical signal (Hilbert transform) were then extracted for each EEG channel. For each pair of electrodes, the PLV was computed as a long-distance synchronization index on 8-s non-overlapping sliding windows as

(2) $$PLV_{i,j}=\left|\frac{1}{N}\overset{N}{\underset{t=1}{\sum }}e^{i(\phi_{i}\left(t\right)-\phi_{j}\left(t\right))}\right|,$$

where *N* is the number of samples considered in each 8-s window, ϕ is the phase, and | | the complex modulus. Thus, PLV measure equates 1 if the two signals are perfectly phase locked across the whole observed time window and equates 0 if they are totally unsynchronized. For each electrode, the amplitude of the analytic signal (Hilbert transform) was stored as a local synchronization index. Nonparametric permutation testing was used to gauge the statistical significance of the effects of binaural and monaural beats on functional connectivity.

### Fourier transform and iCOH

Coherency (magnitude-squared coherence) between two EEG channels can be defined as the measure of a linear relationship (i.e., correlation) between two signals (in this case, electrodes) at specific frequencies. It is calculated as the cross-spectral density between channels *i* and *j,* normalized by the square root of the multiplication of each of their own auto-spectrums. By projecting the results into the imaginary axis, we rid the signal of both immediate (a phase difference of 0) and anti-phase (phase difference of π) connectivity patterns. The imaginary part of coherence is insensitive to spurious correlations due to volume conduction at the expense of being sensitive to signals’ amplitude (as well as phase) and being unableto disentangle spurious from real immediate connectivity patterns (both in phase and anti-phase; Nolte et al., 2004).

iCOH measures were extracted on 8-s non-overlapping sliding windows (similar to the PLV procedure):

(3) $$iCOH_{i,j}=\overset{f2}{\underset{f=f1}{\sum }}Im\left(\frac{S_{i,j}\left(f\right)}{{\left(S_{i,i}\left(f\right)S_{j,j}\left(f\right)\right)}^{1/2}}\right)\text{with}S_{i,j}\left(f\right)=<x_{i}\left(f\right)x_{j}^{*}\left(f\right)>,$$

where $x_{i}\left(f\right)$ and $x_{j}\left(f\right)$ are the complex Fourier transforms of channels *i* and *j*, respectively, * stands for complex conjugation, <> for the expectation value, $f_{1}$ and $f_{2}$ are the boundary of the considered frequency band, $S_{i,j}\left(f\right)$ is the cross-spectral density between channels *i* and *j*, and Im() is the imaginary part of a complex number. As a long-distance synchronization index, iCOH values were averaged for each pair of electrodes across frequency bins using a tolerance of 1 Hz (e.g., 7 ± 1 Hz). Each electrode’s autospectrum was stored as a local synchronization index. As with PLV and Hilbert Transform, nonparametric permutation testing was used to gauge the statistical significance of the effects of binaural and monaural beats on functional connectivity.

### Cross-frequency interactions

In this context, we consider cross-frequency interactions as activity elicited by either experimental condition (binaural or monaural) that is outside of the frequency range of the beat (either 7 Hz for θ, or 40 Hz for γ). For example, activity in the α frequency band elicited by θ experimental conditions is considered as a cross-frequency interaction (Solcà et al., 2016).

### Neurophenomenological analysis

To explore the relationship between mood (as self-reported by the visual analogue scales) and neural patterns of functional connectivity, each participant’s two highest rated experimental conditions (binaural γ, monaural θ …) were contrasted with the two lowest rated ones for each visual analogue scale. These contrasts were then averaged across participants for each scale (mental relaxation and absorption depth). Again, non-parametric permutation testing was used to gauge the statistical significance of the relations between subjective experience and functional connectivity.

### Functional connectivity: nonparametric statistics

Given the exploratory nature of our study, we decided to use nonparametric permutation testing to maintain the FWER at 5%, as it offers a straightforward solution to the multiple-comparisons problem (Maris and Oostenveld, 2007; Groppe et al., 2011). The critical *t* value was determined for all functional connectivity analysis (PLV, iCOH, autospectrum, Hilbert transform, and neurophenomenology) as follows: (1) the experimental conditions were contrasted with each other (binaural vs monaural control) and each experimental condition was contrasted with baseline, (2) a *t* test was performed at each spatial-spectral point (i.e., electrode at a given frequency), (3) the statistics were normalized using *z* scores, (4) the cluster statistic was considered to be the sum of all *t* values of the cluster members exceeding 3 in absolute value, (5) 1000 permutations of the data were then performed to obtain a distribution of cluster statistics under the null hypothesis and determine the critical values. All randomizations were done for a rejection of the null hypothesis and a control of false alarm rate at *p* < 0.05. We decided to choose this method to correct for multiple comparisons because we are mainly interested in broadly distributed effects (Groppe et al., 2011). To make our inferences more conservative, only contrasts that exhibit at least three significant spatio-spectral points are shown here (i.e., electrodes at a given frequency).

## Results

### FFR

To keep analyses consistent, we performed permutation-based statistics when testing for the FFR’s significance.

### θ Pure tones (396.5 and 403.5 Hz)

**Figure 3.**
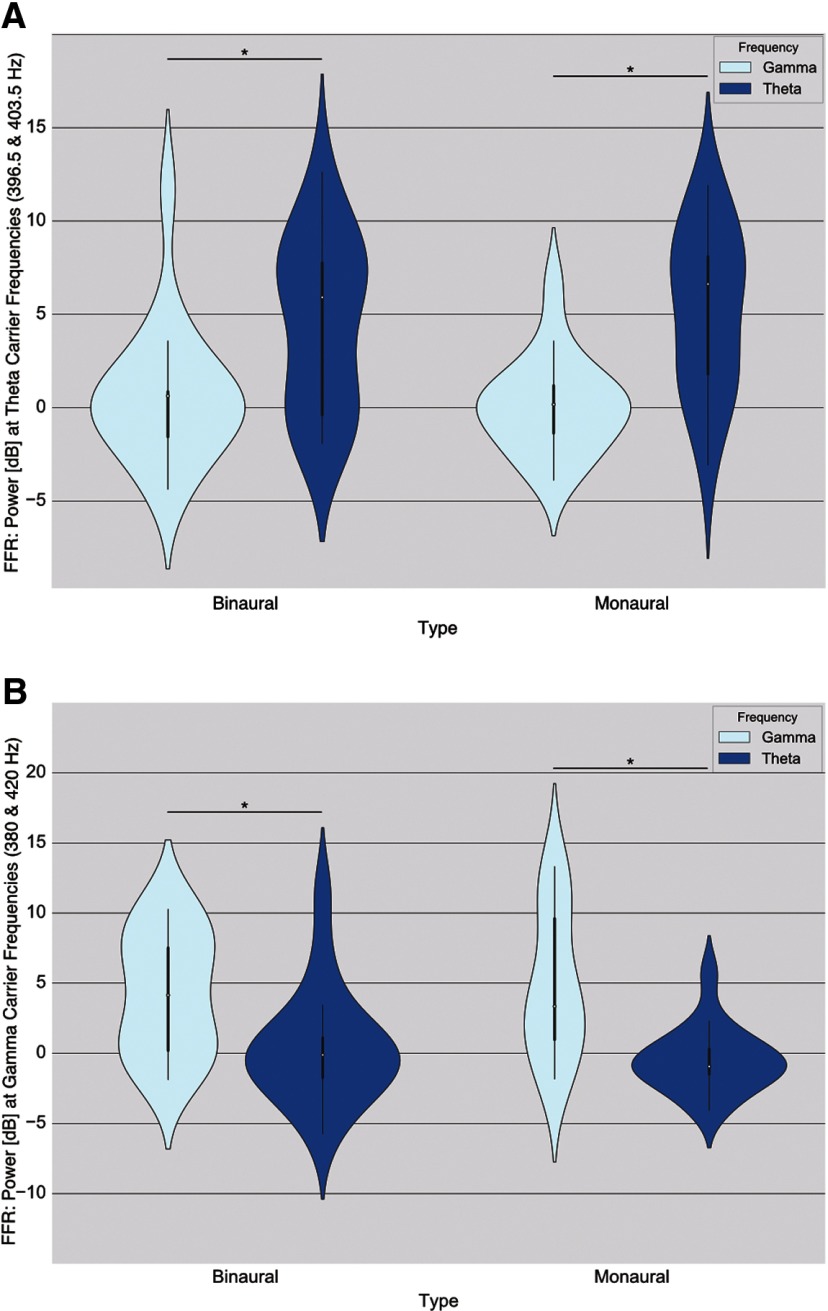
Frequency Following Response (FFR) to carrier pure tones. Plotted here, violin plots with median (white dots), quartile (thick black line), and whisker (thin black line) values. Please note the scale is decibel change from baseline, a logarithmic scale where each 3 dB represents a difference of a factor of 2. Each violin plot represents all participants’ baseline-normalized (dB) averages of the power around a 1-Hz bin (e.g., 396.5 ± 0.5 Hz) at beat carrier frequencies (e.g., 396.5 and 403.5 Hz were averaged together for θ conditions). This power was obtained from the average activity at all channels of each participant. Asterisks above lines linking conditions denote a significant difference between them (*p* < 0.05). ***A***, FFR elicited at θ-carrier frequencies (average of 396.5 and 403.5 Hz). ***B***, FFR elicited at γ-carrier frequencies (average of 380 and 420 Hz).

Both θ binaural and monaural beats elicited an FFR at θ carrier frequencies with no difference between them (average of 396.5 and 403.5 Hz; Fig. 3*A*). There was a main effect of beat frequency (*F* = 34.57, *p* = 0.001)^c^, with no effect of beat type (*F* = 0.004, *p* = 0.96)^c^ nor an interaction between the factors (*F* = 1.169, *p* = 0.292)^c^.

### γ Pure tones (380 and 420 Hz)

γ Binaural and monaural beats elicited an FFR at γ carrier frequencies (average of 380 and 420 Hz; Fig. 3*B*), with no difference between them. There was a main effect of frequency (*F* = 26.648, *p* = 0.001)^d^ with no effect of beat type (*F* = 0.057, *p* = 0.828)^d^ nor an interaction between the factors (*F* = 1.248, *p* = 0.271)^d^.

### ASSR

To keep analyses consistent, we performed permutation-based statistics when testing for the ASSR’s significance.

### θ ASSR (7 Hz)

**Figure 4.**
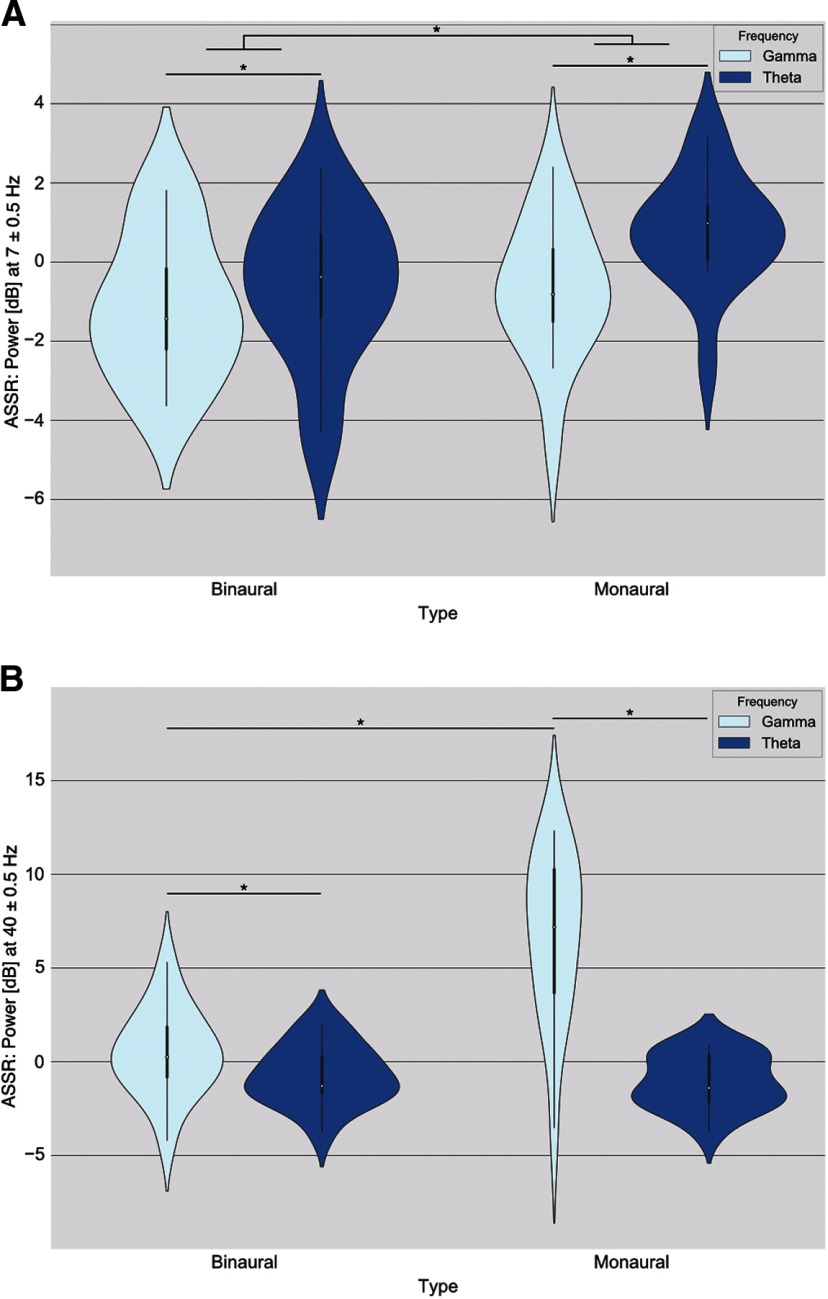
Auditory Steady State Responses (ASSRs) to beat frequency. Plotted here, violin plots with median (white dots), quartile (thick black line), and whisker (thin black line) values. Please note the scale is decibel change from baseline, a logarithmic scale where each 3 dB represents a difference of a factor of 2. Each violin plot represents all participants’ baseline-normalized (dB) averages of the power around a 1-Hz bin (e.g., 7 ± 0.5 Hz) at beat frequencies (either 7 or 40 Hz) obtained from the average activity at all channels for each participant. Asterisks above lines linking conditions denote a significant difference between them (*p* < 0.05). Please note that there was an outlier in these graphs that was taken out for visualization purposes (a participant with data points at around −30 dB). ***A***, Cortical activity elicited at 7 Hz. ***B***, Cortical activity elicited at 40 Hz.

θ Binaural and monaural conditions elicited an ASSR at beat frequency, with monaural beats peaking higher than binaural beats (Fig. 4*A*). There was both a main effect of beat type (*F* = 7.669, *p* = 0.018)^e^ and beat frequency (*F* = 19.263, *p* = 0.001)^e^, with no interaction (*F* = 3.928, *p* = 0.075)^e^.

### γ ASSR (40 Hz)

γ Beats (both binaural and monaural) elicited an ASSR at 40 Hz, with binaural γ eliciting the highest power(Fig. 4*B*). There were main effects of both beat type (*F* = 34.538, *p* = 0.001)^f^ and beat frequency (*F* = 51.933, *p* = 0.001)^f^, as well as an interaction between the two factors (*F* = 44.284, *p* = 0.001)^f^. To further disentangle these differences, three *post hoc* pairwise comparisons were done using Holm’s sequential Bonferroni correction test. The first two comparisons confirmed that monaural γ peaked the highest at 40 Hz when compared with both binaural γ (mean difference = 6.2702, *t* = 7.23 p_corr_ = 0.002)^f^ and monaural θ (mean difference = 7.6589, *t* = 7.68, *p*_corr_ = 0.003)^f^. Binaural γ condition elicited a stronger ASSR than binaural θ at 40 Hz (mean difference = 1.1888, *t* = 2.46, *p*_corr_ = 0.025)^f^.

### Functional connectivity

#### PLV and Hilbert transform amplitude

**Figure 5.**
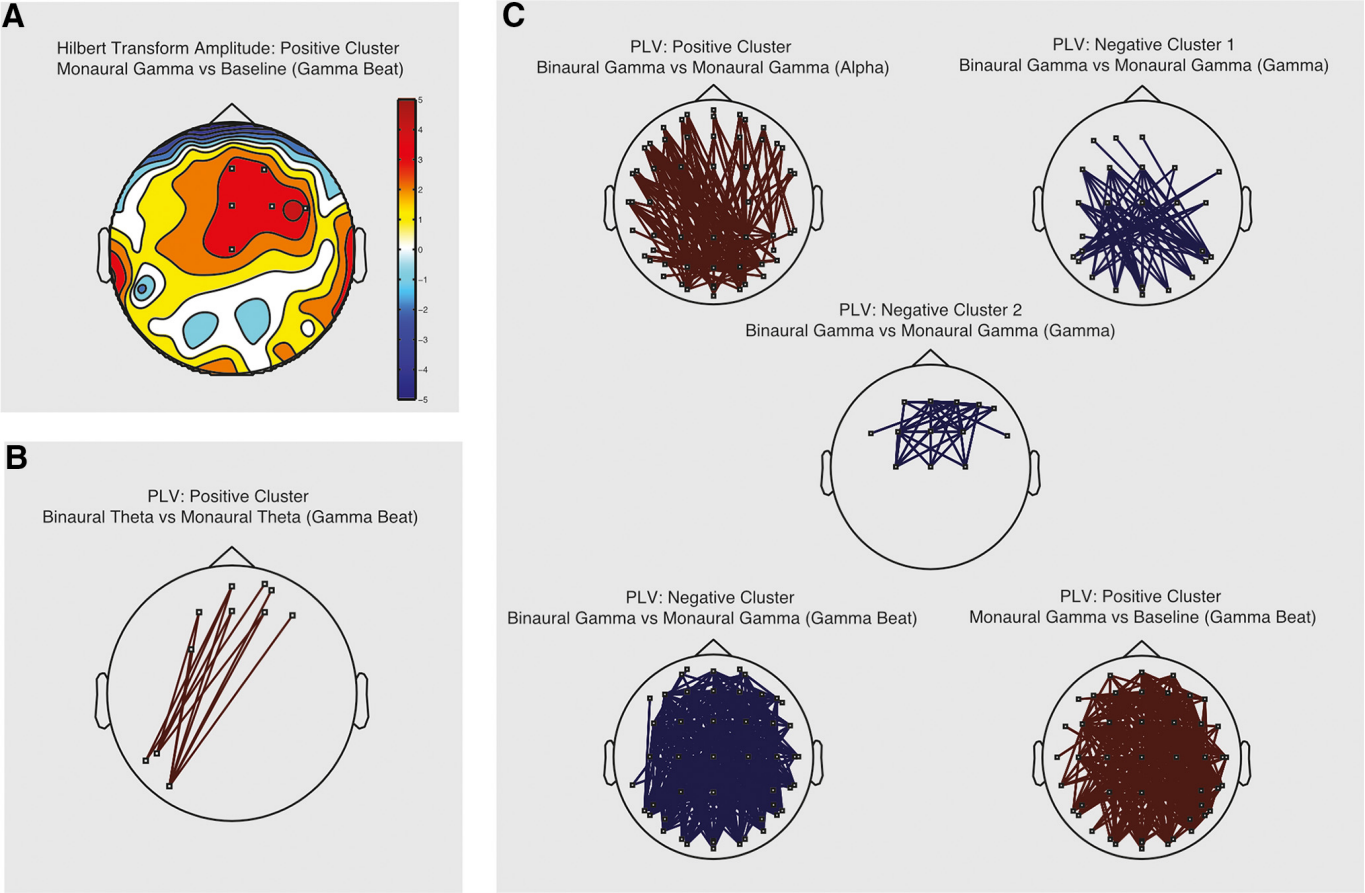
Contrast topographies for Phase Locking Value (PLV) and Hilbert transform amplitude. Topographies were averaged across participants and compared with either baseline or between beat type (binaural vs monaural). Both statistics (Hilbert transform amplitude and PLV) were normalized using *z* scores. We only show contrasts that exhibit at least three significant electrodes (depicted as small white squares). Frequency band limits are as follows: δ (1–4 Hz), θ (5–8 Hz), α (9–12 Hz), β (13–30 Hz), γ (32–48 Hz), θ beat (6–8 Hz), and γ beat (39–41 Hz). ***A***, Hilbert transform amplitude used as a local synchronization index. The color bar indicates *t* values from Student’s test. ***B***, PLV used as an index of long-distance synchronization between electrodes during θ conditions. Red lines indicate a significant positive PLV between two electrodes. ***C***, PLV used as an index of long-distance synchronization between electrodes during γ conditions. Red lines indicate a significant positive PLV between two electrodes while blue lines indicate a negative one.

Both binaural and control conditions elicited within and cross-frequency patterns at long and short ranges. These were dependent on both beat type and frequency. In terms of local synchronization (Hilbert Transform Amplitude), Monaural γ stimulation drove a positive frontoparietal cluster at 40 Hz (γ beat) when contrasted with baseline (Fig. 5*A*; Cluster Statistics = 20.52, *p* = 0.019)^g^. In terms of long-distance synchronization (PLV), we found a positive left-occipital to frontoparietal cluster of activity at 40 Hz (Fig. 4*B*; CS = 49.827, *p* = 0.041)^h^ when contrasting binaural θ with monaural θ experimental conditions. When contrasting binaural γ with monaural γ, we found four clusters of activity: a positive cluster extending around the scalp at α frequency band (Fig. 5*C*, top left; CS = 1043.455, *p* = 0.002)^h^, a negative central-occipital cluster at γ frequency band (Fig. 5*C*, top right; CS = −219.57, *p* = 0.043)^h^; a negative frontal cluster at γ frequency band (Fig. 5*C*, center; CS = −240.17, *p* = 0.028)^h^; and a negative scalp-wise cluster at 40 Hz (Fig. 5*C*, bottom left; CS = −2695.07, *p* = 0.002)^g^. Consistent with the last cluster, the monaural γ condition drove a positive scalp-wise cluster when contrasted with baseline (Fig. 5*C*, bottom right; CS = 2493.34, *p* = 0.004)^h^.

#### iCOH and Fourier transform power

**Figure 6.**
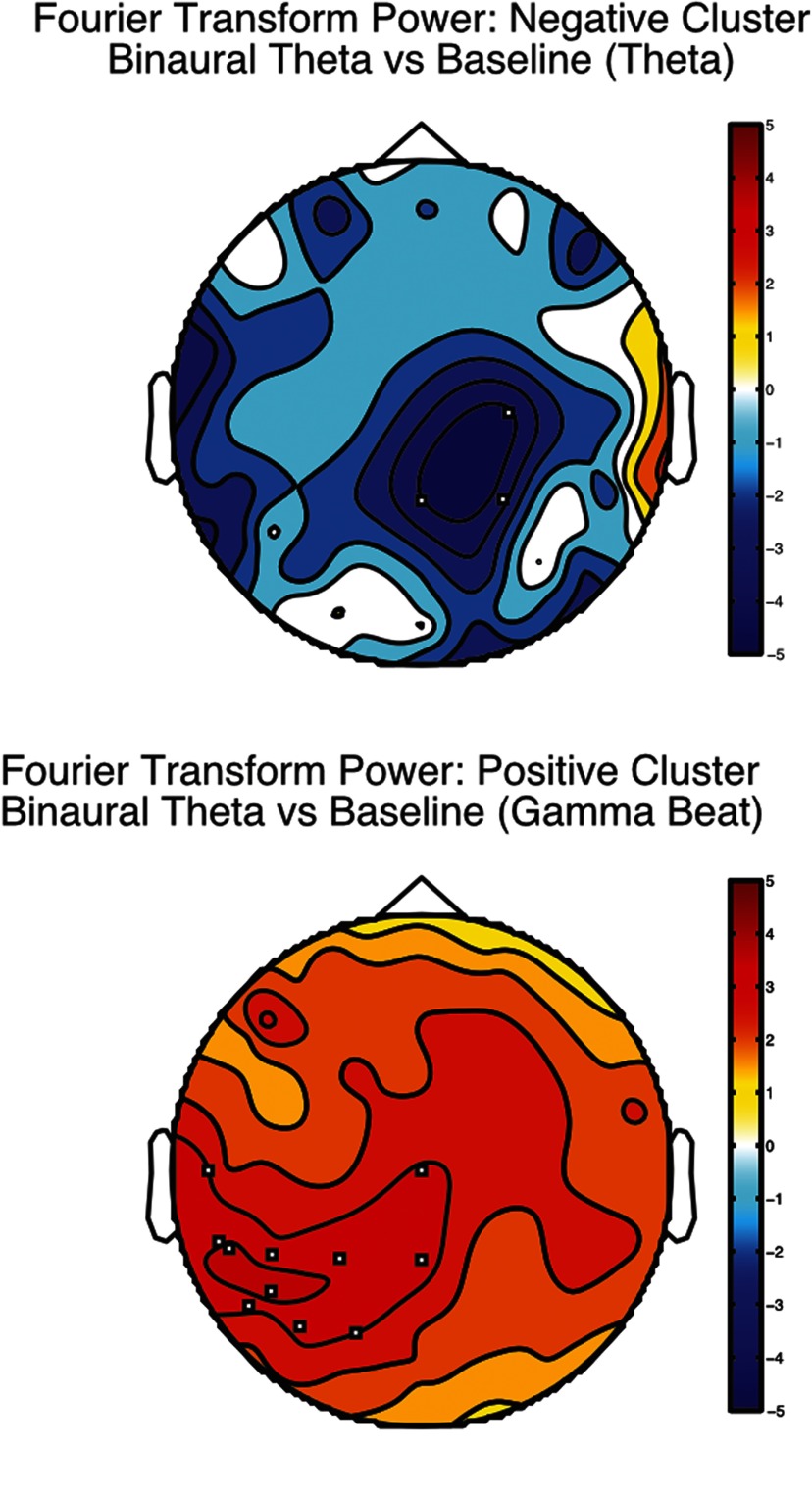
Fourier transform power used as a local synchronization index in θ conditions. Topographies were averaged across participants and compared with baseline. Fourier transform power was normalized using *z* scores. We only show contrasts that exhibit at least three significant electrodes (depicted as small white squares). Frequency band limits are as follows: δ (1–4 Hz), θ (5–8 Hz), α (9–12 Hz), β (13–30 Hz), γ (32–48 Hz), θ beat (6–8 Hz), and γ beat (39–41 Hz). The color bar indicates *t* values from Student’s test. Please note that no Imaginary Coherence (iCOH) contrasts were significant.

As indexed by iCOH and Fourier transform, we only found short distance synchronization elicited by binaural θ conditions. In terms of local synchronization (Fourier power), we found two clusters of activity when contrasting binaural θ condition with baseline: a negative central-parietal cluster of activity at θ frequency band (Fig. 6, top; CS = −11.45, *p* = 0.036)^i^ and a positive left central-temporal cluster at 40 Hz (Fig. 6, bottom; CS = 36.10, *p* = 0.018)^i^. None of the other contrasts reached our criteria for significance (*p* < 0.05 and a cluster of at least three sensors).

#### Neurophenomenological analysis

**Figure 7.**
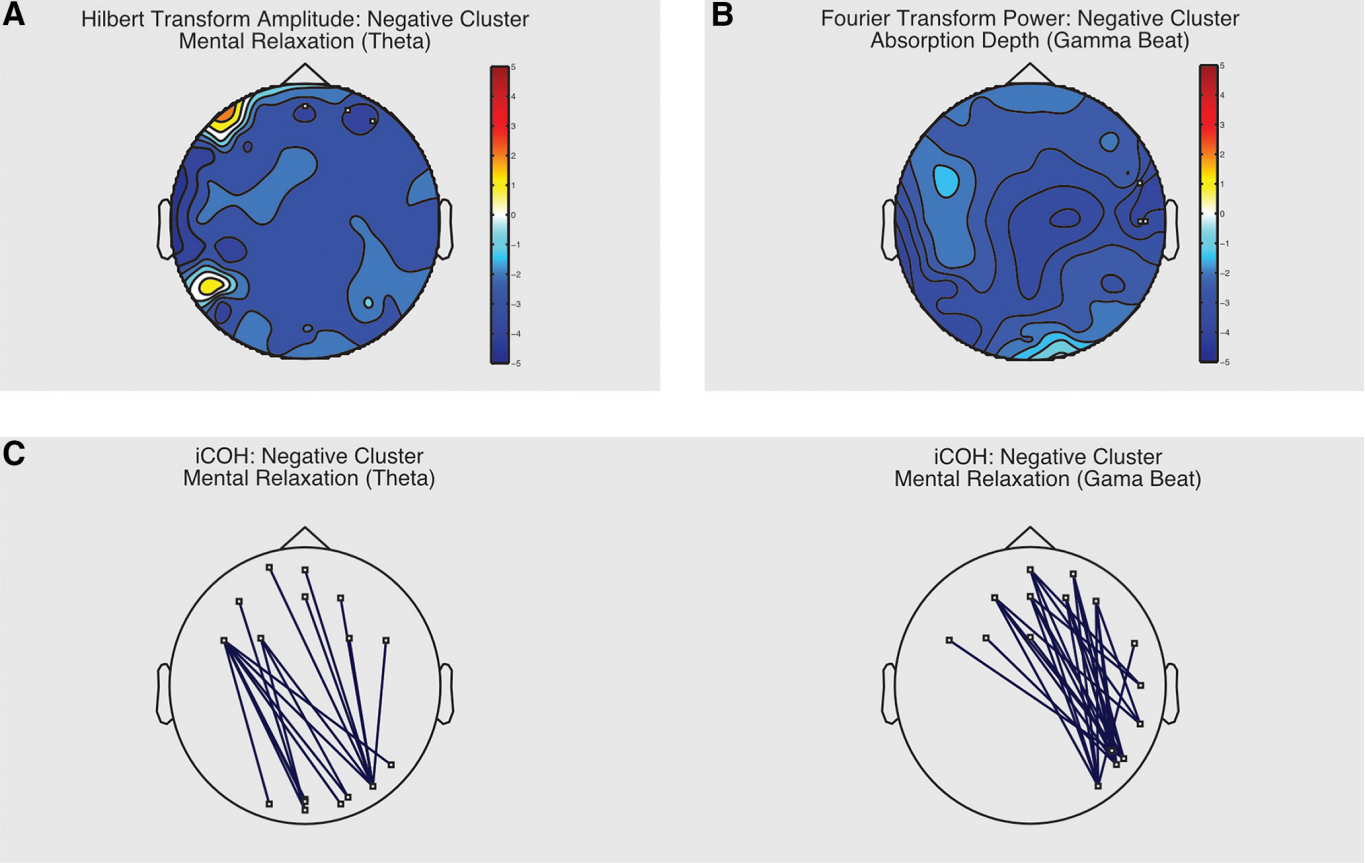
Neurophenomenological analysis: correlates between subjective experience and EEG connectivity patterns. Each participants’ two highest rated (mental relaxation and absorption depth) experimental conditions (binaural γ, monaural θ …) were contrasted with the two lowest rated conditions. These contrasts were then averaged across participants for each separate scale (mental relaxation and absorption depth). All statistics were normalized using *z* scores. We only show contrasts that exhibit at least three significant electrodes here (depicted as small white squares). Frequency band limits are as follows: δ (1–4 Hz), θ (5–8 Hz), α (9–12 Hz), β (13–30 Hz), γ (32–48 Hz), θ beat (6–8 Hz), and γ beat (39–41 Hz). ***A***, Hilbert transform amplitude used as a local synchronization index. The color bar indicates *t* values from Student’s test. ***B***, Fourier transform power used as a local synchronization index. The color bar indicates *t* values from Student’s test. ***C***, Imaginary Coherence (iCOH) used as an index of long-distance synchronization between electrodes. Blue lines indicate a significant negative iCOH between two electrodes.

When taking into consideration individual differences due to subjective experience, we find neural connectivity patterns associated with high absorption depth and mental relaxation that are consistent across participants. When contrasting each participants’ two highest and two lowest rated experimental conditions in terms of mental relaxation, we found one negative frontal cluster of local activity (Hilbert transform; Fig. 7*A*) at θ frequency band (CS = −9.51, *p* = 0.026)^j^, and a negative long-range frontocentral to occipital cluster of activity (iCOH) at the same frequency band (Fig. 7*C*, left; iCOH: CS = −61.95, *p* = 0.034)^j^. We also found a frontal to right-temporal-occipital negative cluster of activity at 40 Hz (Fig. 7*C*, right; iCOH: CS = −97.33, *p* = 0.043)^j^. On the other hand, we also contrasted the two top experimental conditions in which a given participant rated absorption depth the highest with the experimental conditions in which they rated absorption depth the lowest. We found a right temporal negative cluster of local (Fourier power) activity at 40 Hz (Fig. 7*B*; CS = −10.17, *p* = 0.05)^j^.

#### Visual analogue scales

There was no effect from auditory stimulation on subjective ratings (Fig. 2). To keep analyses consistent throughout the manuscript, we performed permutation-based statistics. There were no differences between the five levels of the factor condition (baseline, binaural γ, binaural θ, monaural γ, monaural θ) neither in mental relaxation (*F* = 1.698, *p* = 0.158)^a^ nor absorption depth (*F* = 1.313, *p* = 0.291)^b^. These suggest that subjective experience related to each experimental condition was not different from baseline nor from each other.

## Discussion

Here, we asked whether binaural beats are able to elicit neural entrainment, and modulate mood, in a specific fashion compared with a control rhythmic stimulus. To do so, we used a passive, single-blind listening task where participants were exposed to both binaural and monaural control conditions while we recorded their electrical brain activity and mood self-reports. By comparing activity between binaural and monaural control conditions at different levels (subcortical, cortical and self-reports), we found that binaural beats did entrain the brain, but the control condition did so more strongly, with none of them showing an effect on mood. Furthermore, while distinct functional connectivity patterns emerged for both binaural and monaural beats at different frequencies, these are not consistent with previous literature and are not related to participants’ self-reported mood.

### Binaural and monaural beats elicit subcortical responses at carrier frequencies

Although it is commonly agreed that binaural beats originate in the brainstem (Wernick and Starr, 1968; Oster, 1973; Moore, 2012), to the best of our knowledge, we are the first ones to investigate this particular stimulus at subcortical levels using EEG. As we predicted, both experimental conditions (θ and γ), regardless of beat type, elicited an FFR at the pure tone frequencies, with no difference between monaural and binaural beats. This is consistent with the existing auditory brainstem response literature, where the generated subcortical responses are found to have a close spectrotemporal structure to the patterns of an acoustic stimulus, such as speech syllables (Skoe and Kraus, 2010; Lehmann and Schönwiesner, 2014). Furthermore, given our choice of carrier frequencies (around 400 Hz), it is very unlikely these responses have a cortical origin (Coffey et al., 2016). The lack of difference between beat types suggests that both stimuli are processed in a similar way at the subcortical level.

### Monaural beats elicit higher cortical entrainment at the beat frequency than their binaural counterparts

Both beat types entrained the brain at their beat frequencies, with monaural conditions eliciting the highest response when compared with binaural conditions. In terms of θ beat frequency, both Jirakittayakorn and Wongsawat (2017) and Karino et al. (2006) found similar entrainment using θ binaural beats with exposure times between five and 10 min. Following Garcia-Argibay et al., 2019a)’s conclusions, relatively long exposure time and non-masked binaural beats (i.e., not using white or pink noise to mask them) seem to optimize the responses to the beats. In terms of γ beat frequency, we successfully replicated previous studies (Schwarz and Taylor, 2005; Draganova et al., 2008; Ross et al., 2014): both binaural and monaural γ beats entrain cortical activity at 40 Hz, but binaural beats elicit less power at the beat frequency. One possible explanation as to why binaural beats elicit less power than monaural beats is that the entrainment we measure at the cortical level might be caused by the perceived rhythmicity, and not the binaural beat itself. Subjects’ tended to report that the modulation (i.e., the beat) intensity in binaural beats was weaker than that of the monaural beat. The ASSR correlates with stimulus’ loudness (Lins and Picton, 1995; Picton et al., 2007; Van Eeckhoutte et al., 2016), which might explain the difference in ASSR power in the frequency domain. Furthermore, we both rms normalized and carefully calibrated our stimuli, precluding loudness as an explanatory factor for the difference in ASSR power. Using proper statistical and experimental control, we have shown that binaural beats entrain the cortex more weakly than other non-binaural rhythmic stimuli, such as monaural beats.

### Binaural and monaural beats fail to modulate mood

Echoing previous reports, we did not find evidence of binaural beats, nor monaural beats, modulating cognitive states or mood: López-Caballero and Escera (2017) found no emotional regulation due to binaural beats as indexed by changes in heart rate and skin conductance, while Gálvez et al. (2018) found no modulation of anxiety as indexed by the state anxiety inventory (SAI). This stands in contrast with other reports where cognitive performance and mood were successfully modulated by binaural beats (Le Scouarnec et al., 2001; Padmanabhan et al., 2005; Wahbeh et al., 2007; Reedijk et al., 2013, 2015; Isik et al., 2017; Garcia-Argibay et al., 2019a).

### Both beat types elicit differential short-range connectivity patterns

Monaural and binaural beats affect short range electrode level connectivity patterns differentially. We only found a significant short-range effect in monaural γ and binaural θ conditions, which suggests that both beat type and frequency are non-trivial parameters of stimulation. Furthermore, due to both binaural and monaural beats producing such short-range effect, we can rule out conclusions such as this activity being a by-product of sustained listening or binaural integration. Our γ findings are in accordance with those from Becher et al. (2015). Using both intracranial and scalp EEG, they found peak EEG power at 40 Hz (γ beat) at the scalp electrodes using the power of the envelope of the signal (i.e., the power of the Hilbert transform). They found a similar effect in temporo-lateral intracranial electrodes, suggesting this entrainment originates in auditory cortices. Furthermore, they found a significant decrease in EEG power at 5 Hz (θ frequency) in temporo-basal anterior and posterior areas, which might explain the local activity in our participants. This activity could be in line with a dipole from auditory cortices pointing upwards, which suggests there is only one active cortical source. On the other hand, binaural θ conditions elicited a positive parietal cluster at 40 Hz (see cross-frequency section). The functional meaning (if any) of these short-range patterns remains unclear as we found no difference in participants’ self-reports.

### Both beats elicit long-range connectivity patterns indexed only by PLV

To investigate long-range connectivity, we used two different but complementary statistics: the imaginary part of coherence and the PLV. iCOH gets rid of all interactions that have zero to very small time delays, while the PLV quantifies how consistent phase differences are between electrodes. We only found functional connectivity patterns indexed by PLV. Because of this, it is unclear whether these patterns are due to one source being propagated around the scalp, or there are multiple sources active with an almost zero-time delay between them (Nolte et al., 2004). We see differential effects between beat types and frequencies, as well as cross-frequency interactions (discussed in the next section). Only γ experimental conditions elicited within frequency activity: Monaural γ elicited a cluster of scalp-wise connectivity that is not consistent with previous research. Using intracranial electrodes, Becher et al. (2015) showed phase desynchronization at mediotemporal areas using a 40 Hz monaural beat, whereas we found scalp-wise synchronization using a very similar stimulus. Furthermore, Schwarz and Taylor (2005) showed that there was a delay of several milliseconds in the activity elicited across the fronto-occipital axis, using a 40 Hz monaural beat, suggesting multiple cortical sources of activity. Because the iCOH analysis did not reveal significant connections between electrodes, it is unclear whether the phase differences Schwarz and Taylor (2005) report are due to volume conduction or the connectivity patterns we found are caused by multiple, but tightly synchronized, brain regions. Because we did not find any difference in subjective reports, the functional meaning (if any) of this activity remains unclear.

### Binaural beats elicit cross-frequency connectivity patterns

Binaural θ conditions elicit a front to back, cross-frequency connectivity pattern at γ beat frequency (40 Hz) while binaural γ elicits a widespread connectivity pattern at α frequency. In line with our results, several groups have found binaural beats eliciting activity outside of the frequency range of the beat. Despite this, these findings do not seem to be consistent: using θ binaural beats, Gao et al. (2014) found a decrease in relative β power over left temporal areas and Ioannou et al. (2015) report no significant difference from θ binaural beats at other frequency bands.

We found that binaural θ beats elicit activity at higher frequencies (40 Hz), while binaural γ beats elicit activity at lower frequencies. This cross-frequency coupling (low frequency driving a higher frequency and a higher frequency driving a lower frequency) could be evidence for large-scale integration being enhanced by binaural beats. Varela et al. (2001) argue that slower rhythms (such as θ) provide a temporal framing for faster oscillations. For example, γ oscillations are thought to leverage this slower temporal framing during successive cognitive moments of synchronous assemblies where memory is consolidated (Osipova et al., 2006; Burke et al., 2013; Lisman and Jensen, 2013). We did not investigate cognitive processes directly, but there is evidence that binaural beats impact memory as a function of beat frequency, β frequencies seems to enhance it (Kennerly, 1994; Lane et al., 1998; Beauchene et al., 2016; Gálvez et al., 2018) while θ frequencies have an inconsistent effect (negative in some cases: Beauchene et al., 2016; Garcia-Argibay et al., 2019b; and positive in others: Ortiz et al., 2008). In our specific experiment, our stimuli failed to modulate mood as self-reported by participants, but these cross-frequency interactions might provide a framework explaining why binaural beats are able to modulate cognitive performance in other reports.

### Individual differences shed light on connectivity patterns associated with specific cognitive states

We found a consistent pattern of deactivation and desynchronization related to participants’ self-reports of mental relaxation and absorption depth. High mental relaxation was associated with θ frequencies in a frontal cluster of local desynchronized activity, and with a front to back desynchronization of activity at both θ and γ indexed by iCOH. This suggests this activity is robust and not due to volume conduction. Absorption depth, on the other hand, was associated with one cluster of activity around temporal areas. Changes in the anterior and frontal midline in θ power have been related to emotionally positive states (Aftanas and Golocheikine, 2001), and meditation-related states (Baijal and Srinivasan, 2010). Baijal and Srinivasan (2010) found a similar deactivation pattern in parietal and occipital areas accompanied by frontal θ activation associated with meditative states. On the other hand, Hinterberger et al. (2014) found similar central and parietal γ deactivation patterns during meditative tasks. Taking all this information together, our functional connectivity results point at a state similar to meditation characterized by heightened mental relaxation and absorption depth. Despite this, we were not ableto relate this specifically to any of our experimental conditions.

### Limitations and future directions

Though several of our findings are consistent with existing literature, we acknowledge that we only recruited 16 people and that these findings should be replicated with higher sample sizes. We instructed participants to close their eyes during the whole experiment, which might have not been ideal, especially because a couple of participants reported high drowsiness and two reported falling asleep. Furthermore, we did not have a specialist check our participants for any hearing loss. This could be a co-founding variable. Our design, however, was a withindesign: each participant was exposed to the four experimental conditions. Because participants were only compared against themselves (statistically speaking), thisco-founding effect remained constant for each participant. Future binaural beats studies should look at different ways of indexing connectivity at both scalp and source level (Solcà et al., 2016). The study of binaural beats will also greatly benefit from the transition of a mass univariate statistical framework (such as the one used here) to a multivariate statistical framework (McIntosh and Mišić, 2013). Fields such as graph theory present promising opportunities to determine the characteristics of cortical networks and summarizing large numbers of data points into a few statistics to truly understand how binaural beats affect the brain (Ioannou et al., 2015; Ala et al., 2018). On a more technical note, we did not alternate the polarity of the stimulus of the binaural beats (a common practice in the auditory brainstem response literature), which might have affected the brainstem responses we found. Sometimes, noise from the audio transducer or the cochlear microphonic (a potential believed to be generated primarily by outer hair cells; Santarelli et al., 2006) can bleed into the EEG electrodes, introducing artifactual responses into the EEG trace. To minimize this, one can alternate the polarity of the stimulus between each presentation, i.e., by presenting the original stimulus followed by a version of the stimulus that has been multiplied by minus one and repeating this over and over. After this, the EEG responses to both polarities can be either added or subtracted together (each method has its advantages and disadvantages). For an in-depth discussion of this, see Skoe and Kraus (2010). The transducer with which the stimuli were presented to the participants was magnetically shielded, a procedure that is known to minimize stimulus artifacts (Skoe and Kraus, 2010). Furthermore, Skoe and Kraus (2010) report that “[their] results have been internally replicated with single-polarity stimuli,” supporting our claim that the responses we report here come from the brainstem and are not artifactual.

Binaural beats have been long used in psychoacoustics, although the claims and studies relating their cognitive effects are more recent. A great deal of confusion subsides regarding their latter use. As Garcia-Argibay et al. (2019a) concluded, there are several mediating variables, such as beat frequency, exposure time or stimulus masking, that are not always clearly reported. Furthermore, we conclude that there are two important factors that are usually considered as trivial: proper EEG analysis and the use of a proper control condition. Future binaural beats studies should be mindful of these variables and report them accordingly. Several studies that did not find any entrainment to the beat frequency of binaural beats (López-Caballero and Escera, 2017) did not use standard normalization practices (for an in-depth discussion of these procedures, see Cohen, 2014, Chapter 18). The human EEG spectrum exhibits a 1/f power scaling (similar to pink noise). By properly normalizing data using a baseline condition, we ensure that all data (i.e., the different frequency bands) will have the same scale, and we are appropriately disentangling background and task-unrelated dynamics (Cohen, 2014). Researchers should be mindful of the analysis approach they are taking, as well as using an appropriate control condition to truly elucidate whether binaural beats are a special kind of stimulus or their advantages are due to stimulus properties (such as the rhythmicity in the signal).

Future studies should carefully choose exposure time, the performance or mood measurement and the frequency of the beat. As Garcia-Argibay et al. (2019a) concluded, higher exposure times are associated with larger effect sizes. Nevertheless, whether several sessions will present an increased entrainment and performance/mood boost, and whether there are carryover effects that are sustained even after stimulation ceases, are still open questions. Binaural beats have previously been reported to modulate memory and attention performance (Ortiz et al., 2008; Reedijk et al., 2015), as well as anxiety (Isik et al., 2017) and analgesia (Zampi, 2015). Our findings provide one plausible base explanation as to why memory and attention performance could be modulated by binaural beats (i.e., binaural beats elicit cross-frequency interactions). Future studies should focus on measuring cognitive performance on both attention and memory tasks, and mood regulation related to anxiety and pain perception. Participants should be exposed to the stimulation both before and after the task (Garcia-Argibay et al., 2019a). Finding neural correlates of binaural beats that uniquely correlate with cognitive performance in attention and memory tasks could help better elucidate whether binaural beats can be used for cost-effective cognitive enhancement. Finally, the choice of frequency is not trivial: α, β, and γ were reported to provide positive effects in memory tasks, while θ frequency seems to hinder effects in most cases (Garcia-Argibay et al., 2019a).

## Conclusions

Using a factorial experimental design and a single-blind, passive listening task, we aimed to elucidate the impact of binaural beats on the brain. We did not find evidence for binaural beats modulating mood or entraining the brain more strongly than “non-binaural” beats. We did find, however, that binaural beats elicited differential patterns of connectivity, compared with the monaural beat control. Whether these connectivity patterns have a functional meaning (in terms of cognitive enhancement and mood modulation) remains an open question. The present research shares a useful framework for further exploring the mechanisms and efficacy of sound-based mood regulation practices. By using a neuroscientific lens with statistical and scientific rigor at its core, we can study these “alternative” practices to ensure the general public makes informed, evidence-based, decisions.

## Synthesis

Reviewing Editor: Tatyana Sharpee, The Salk Institute for Biological Studies Decisions are customarily a result of the Reviewing Editor and the peer reviewers coming together and discussing their recommendations until a consensus is reached. When revisions are invited, a fact-based synthesis statement explaining their decision and outlining what is needed to prepare a revision will be listed below. The following reviewer(s) agreed to reveal their identity: Roland Ferger.

This manuscript addresses Binaural Beats, an auditory illusion arising when two neighboring frequencies as presented to each ear separately. Binaural Beats have been implied in mood changing and cognitive enhancement by scientific and non-scientific publications. The authors have designed a "sham" controlled study do investigate the effects of Binaural Beats on activity at different levels of processing. The experimental design and thorough, yet straight forward, analysis allow a scientifically proper insight into effects of Binaural Beats at these levels. Due to the study design, the conclusions that can be drawn about effects on mood and cognitive performance are very limited. The authors address this limitation with the proper caution and outline possible follow-up studies in the future directions section.

Statistics:

The phrase "to further disentangle these differences" is used to justify post hoc t-tests of all tested conditions, without giving the reader a functional relevance or methodological reason and then just report significant differences. This is not a good practice.

Reviewer 1:

It is not clear if the same (e.g. the lower) of two frequencies was always presented to the same ear (e.g. left). This could be clarified in the Materials & Methods - Stimuli section. Is this what is meant in the discussion by "we did not alternate the polarity of the stimulus of binaural beats"?

In my opinion, the self-reported data - shown only in the visual abstract as Fig. 4 (bottom right) - should also be reported as figure within the main body of the manuscript, even though "no effect" was found as mentioned at several positions throughout the text.

To the "Violin"-plots: a) "Beat Frequency" instead of "Frequency" as legend title might help the reader to understand these plots slightly faster - in light of several frequencies playing a role in this manuscript. b) In Fig 3b, a y-tick at zero should be shown, because of the plotted change in dB. Maybe consider a closer spacing between y-ticks in both plots of Fig 3.

Reviewer 2:

1. The sham stimulus could be designed incorrectly and might not allow the comparison the authors want to make. The authors insufficiently describe why they chose the sham stimulus and how exactly it was constructed. If they actually just superimposed the 2 frequencies, they in my opinion created amplitude modulated binaural beats (AMBB) (see Dietz (...) McAlpine 2010, https://doi.org/10.1073/pnas.1309712110). The auditory system is specialized to pick up AMBBs, so it's no wonder that they find entrainment in their EEG recordings. If that was on purpose the authors never mentioned it. But the way it was written I think it was not on purpose and they didn't think of that. I personally feel that a sham control that does not elicit entrainment (maybe white noise?) would enable the authors to make better claims about the entrainment and mood altering effects of binaural beats.

2. The authors never mention why binaural beats are a great stimulus. It is effective because it creates the illusion of a third sound lateralizing between the left and right ear of the listener. If chosen right, 1 second of binaural beats will contain all possible interaural time differences of the listener in it, which makes it a fantastic tool for sound source localization experiments. The authors mention in line 93 that hearing loss was self reported by participants. This is a red flag, as a hearing loss prohibits humans from experiencing the lateralization of the binaural beat. If no audiologist checked the participants for normal hearing, the results are confounded, as a 0 - 20dB hearing loss is often not recognized by patients themselves. It was also never mentioned if the earphones were calibrated properly (line 38), so the authors were not careful about making sure that the listeners are exposed to the stimulus properly and can actually perceive it.

3. This manuscript glances over some important details which need to be mentioned in the methods section and could benefit from a more extensive and more descriptive results section.
